# TBX20 Contributes to Balancing the Differentiation of Perivascular Adipose-Derived Stem Cells to Vascular Lineages and Neointimal Hyperplasia

**DOI:** 10.3389/fcell.2021.662704

**Published:** 2021-06-02

**Authors:** Yongli Ji, Yuankun Ma, Jian Shen, Hui Ni, Yunrui Lu, Yuhao Zhang, Hong Ma, Chang Liu, Yiming Zhao, Siyin Ding, Meixiang Xiang, Yao Xie

**Affiliations:** ^1^Department of Cardiology, The Second Affiliated Hospital of Zhejiang University School of Medicine, Hangzhou, China; ^2^Department of Endocrinology, The Second Affiliated Hospital of Zhejiang University School of Medicine, Hangzhou, China

**Keywords:** Tbx20, perivascular adipose-derived stem cells, SMC differentiation, EC differentiation, neointimal hyperplasia

## Abstract

**Background:**

Perivascular adipose-derived stem cells (PVASCs) can contribute to vascular remodeling, which are also capable of differentiating into multiple cell lineages. The present study aims to investigate the mechanism of PVASC differentiation toward smooth muscle cells (SMCs) and endothelial cells (ECs) as well as its function in neointimal hyperplasia.

**Methods:**

Single-cell sequencing and bulk mRNA sequencing were applied for searching key genes in PVASC regarding its role in vascular remodeling. PVASCs were induced to differentiate toward SMCs and ECs *in vitro*, which was quantitatively evaluated using immunofluorescence, quantitative real-time PCR (QPCR), and Western blot. Lentivirus transfections were performed in PVASCs to knock down or overexpress TBX20. *In vivo*, PVASCs transfected with lentivirus were transplanted around the guidewire injured femoral artery. Hematoxylin–eosin (H&E) staining was performed to examine their effects on neointimal hyperplasia.

**Results:**

Bulk mRNA sequencing and single-cell sequencing revealed a unique expression of TBX20 in PVASCs. TBX20 expression markedly decreased during smooth muscle differentiation while it increased during endothelial differentiation of PVASCs. TBX20 knockdown resulted in the upregulation of SMC-specific marker expression and activated Smad2/3 signaling, while inhibiting endothelial differentiation. In contrast, TBX20 overexpression repressed the differentiation of PVASCs toward smooth muscle cells but promoted endothelial differentiation *in vitro*. Transplantation of PVASCs transfected with TBX20 overexpression lentivirus inhibited neointimal hyperplasia in a murine femoral artery guidewire injury model. On the contrary, neointimal hyperplasia significantly increased in the TBX20 knockdown group.

**Conclusion:**

A subpopulation of PVASCs uniquely expressed TBX20. TBX20 could regulate SMC and EC differentiation of PVASCs *in vitro*. Transplantation of PVASCs after vascular injury suggested that PVASCs participated in neointimal hyperplasia via TBX20.

## Introduction

Adipose tissue is important for cardiovascular homeostasis. Adipose tissue is initially thought as an organ for energy storage but was recently discovered to be rich in adipose-derived stem cells (ASCs). These cells express the surface markers of adult mesenchymal stem cells and can give rise to multiple lineages, such as cardiomyocytes, endothelial cells, smooth muscle cells, osteoblasts, chondrocytes, and adipocytes (Gimble et al., 2007; Bunnell et al., 2008). Previous studies mainly focused on subcutaneous and abdominal adipose tissue and their inner ASCs for their easy access and abundance in number (Pitrone et al., 2017). Little attention has been paid to characterizing the resident stem cells in perivascular adipose tissue (PVAT), named PVASCs. PVAT locates outside the adventitial layer of systemic blood vessels except cerebral vessels and participates in regulating vascular function (Rajsheker et al., 2010). In a murine femoral artery guidewire injury model, transplantation of PVAT accelerated neointimal hyperplasia whereas subcutaneous adipose tissue inhibited neointimal hyperplasia (Manka et al., 2014). Gu et al. (2019) demonstrated that PVASCs transferred to the perivascular side of grafted vein could migrate to neointima and subsequently differentiate into SMCs. Pan et al. (2019) confirmed that PVASCs from old mice could aggravate neointimal hyperplasia compared to those from the young in a murine guidewire femoral artery injury model. However, the mechanism of PVASCs differentiation toward SMCs is still unclear.

The differentiating capacity of ASCs from other depots toward ECs and SMCs *in vitro* has been verified (Ma et al., 2017; Arderiu et al., 2019). SMC differentiation is associated with a high expression of SMC-specific proteins, such as smooth muscle α-actin (α-SMA), Calponin, and SM22α (Mack, 2011). Transforming growth factor-β1 (TGF-β1) was commonly applied for SMC differentiation in multiple pluripotent stem cells (Owens, 1995). In the Smad-dependent signaling pathway, TGF-β1 activates the TGF-β receptor, leading to the phosphorylation of Smad2/3. The phosphorylated Smad2/3 forms a complex with Smad4 and translocates to the nucleus, subsequently modulating target gene expression (Derynck and Zhang, 2003). ECs are generally identified through a variety of specific markers, such as CD31, VE-cadherin, VEGFR1, and VEGFR2. Increasing evidence indicated that ASCs from other depots could give rise to ECs *in vitro* and had proangiogenic effects in the hindlimb ischemia model (Planat-Benard et al., 2004; Hutchings et al., 2020). However, the differentiation of PVASCs toward ECs has not been investigated yet.

TBX20 is a crucial transcription factor for the heart, eyes, ventral neural tube, and limbs during embryonic development. Its deficiency could cause congenital heart disease (Meins et al., 2000). In humans, mutations in TBX20 were associated with a complex spectrum of developmental abnormalities, including defects in septation, chamber growth, and cardiomyopathy (Kirk et al., 2007). TBX20 knockout in mice results in embryonic lethality around E10.5 (Stennard et al., 2005). Overexpression of TBX20 in adult cardiomyocytes promoted proliferation and improved the myocardial infarction outcomes in mice (Xiang et al., 2016). TBX20 overexpression enhanced cardiomyogenic differentiation and inhibited osteogenic differentiation in human subcutaneous adipose-derived MSCs (Neshati et al., 2018; Mollazadeh et al., 2019). In endothelial cells, TBX20 upregulated peroxisome proliferator-activated receptor-γ, suppressed the generation of reactive oxygen species, and decreased the expression of adhesion molecules in response to oxidized low-density lipoproteins (Shen et al., 2013). In zebrafish endothelial development, transient knockdown of TBX20 impaired angiogenesis (Meng et al., 2018).

Our bulk RNA-seq confirmed that PVASCs uniquely expressed TBX20. Single-cell RNA-seq analysis revealed a unique subpopulation of PVASCs which highly expressed TBX20. These findings led us to investigate its role in PVASCs. In the present study, we verified that TBX20 expression significantly changed during PVASC differentiation toward SMCs and ECs. Furthermore, we confirmed that TBX20 regulated both SMC and EC differentiation simultaneously *in vitro*. In a mouse femoral artery guidewire injury model, overexpression of TBX20 in PVASCs decreased neointimal hyperplasia after vascular injury. These findings highlighted the importance of TBX20 for PVASC differentiation and vascular neointimal hyperplasia.

## Materials and Methods

### Experimental Animals

All animal experiments were approved by the Animal Ethics Committee of Zhejiang University in accordance with the Guide for the Care and Use of Laboratory Animals. C57/BL6 mice were purchased from Shanghai Model Organisms Center. All animals were fed a chow diet in a 12-h light/dark environment at 25°C in the Animal Care Facility of the Second Affiliated Hospital, Zhejiang University School of Medicine.

### Isolation and Culture of Adipose-Derived Stem Cells

ASCs were isolated from mouse subcutaneous, abdominal, and perivascular adipose tissue, as described previously (Gu et al., 2019). Briefly, adipose tissue was cut into 1-mm^3^ small pieces, which was then digested with 2 mg/mL collagenase type I (Gibco) and 1 mg/mL Dispase II (Sigma) in Hanks’ balanced salt solution (HBSS) at 37°C for 30–45 min with occasional vortex. The digestion was stopped by DMEM/F12 with 10% FBS and subsequently passed through a 100-μm filter followed by a centrifugation at 300 g for 5 min. Cell precipitates were resuspended in a stem cell culture medium as the following: Minimal Essential Medium-α (Gibco) with 15% fetal bovine serum (Gibco), 10 ng/mL recombinant human leukemia inhibitory factor (Sigma), 5 ng/mL recombinant human FGF (R&D), 2 mmol/L L-glutamine (Sigma), 100 U/mL penicillin, and 100 mg/mL streptomycin (Gibco); they were then incubated in a 5% CO_2_ incubator. The cells were passaged at a ratio of 1:3 every 2 or 3 days. The medium was refreshed every 2 days.

### Cell Identification

PVASCs at passages 3–6 were analyzed using a fluorescence-activated cell sorter (FACS). Briefly, cells were incubated for 30 min at 4°C with the following antibodies: Sca1-Percp (abcam); CD105-FITC (abcam); CD90 (Invitrogen); CD45-FITC (abcam); and CD31-PE (BD Biosciences). The cells without any staining were served as blank. Flow cytometry was performed on a BD flow cytometer. FCS files were exported and analyzed using FlowJo V10 software.

The adipogenic differentiation medium, chondrogenic differentiation medium, and osteogenic differentiation medium were purchased from CHEM. PVASCs were seeded on gelatin-coated plates and then cultured with a specific differentiation medium and maintained for 2 weeks. The medium was changed every 3 days. After 2 weeks, cells were stained with Alcian Blue, Oil red O, or Alizarin Red S(CHEM) following the manufacturers’ instructions, respectively, to assess adipogenic, chondrogenic, and osteogenic differentiation.

PVASCs are positive for Sca1, CD90, and CD105 and negative for CD45 and CD31 (Supplementary Figures 1A,B). PVASCs were capable of differentiating into adipocytes, chondrocytes, and osteoblasts, respectively (Supplementary Figures 1C–E).

### Cell Differentiation

PVASCs from passages 3 to 6 were used for this study. For smooth muscle differentiation, 5 ng/mL Recombinant Mouse TGF-β1 (R&D) was added to induce the cell differentiation. For endothelial cell differentiation, cells were cultured in EGM-2 medium (EGM-2 Bullet kit; Lonza) enriched with growth factors such as VEGF (vascular endothelial growth factor), FGF (fibroblast growth factor), EGF (epidermal growth factor), IGF-1 (insulin-like growth factor), ascorbic acid, hydrocortisone, and heparin, according to instruction provided by the medium manufacturers. Differentiated cells were analyzed by QPCR, Western Blot, and immunofluorescence within 7 days.

### Lentiviral Vector Transduction

Lentiviruses containing full-length cDNA of TBX20 (LV-TBX20) or negative control viruses (LV-NC) were constructed by GeneChem (Shanghai, China). Lentiviral particles containing shRNA against TBX20 (sh-TBX20) or scramble shRNA (sh-scramble) were prepared by GeneChem (Shanghai, China). The lentiviruses were transfected into PVASCs with MOI 50, according to the manufacturer’s protocol. The efficiency of lentiviruses was verified by QPCR and Western Blot analysis after 72 h.

### Western Blot

Protein was extracted from ASCs using RIPA lysis buffer (Beyotime) and then was quantified using a BCA Protein Assay Kit (Thermo Fisher). Ten to fifty microgram of each protein sample was separated via SDS-PAGE and electro-transferred onto PVDF membranes. Following blockade with PBST containing 5% BSA, the membranes were incubated with the following primary antibodies overnight at 4°C: anti-β-Actin (Santa Cruz); anti-TBX20 (sigma); anti-α-SMA (abcam); anti-Calponin (abcam); anti-SM22α (abcam); anti-Smad2 (HuaBio); anti-Smad3 (HuaBio); anti-phospho-Smad2 (HuaBio); anti-phospho-Smad3 (HuaBio); anti-CD31 (HuaBio); anti-VE-cadherin (R&D); and anti-VEGFR1 (HuaBio). The membranes were then washed four times with Tris-buffered saline/0.5% Tween-20 and incubated at 37°C for 1 h with the secondary antibodies (1:3,000; Thermo Fisher). Antigen and antibody complexes were detected by using an ECL Kit (Millipore). Immunoblots were quantified using Image Lab (version 6.0) software.

### Quantitative Real-Time PCR

Total RNA was extracted from cells using the TRIzol reagent (Invitrogen) according to the manufacturer’s instructions. One thousand nanograms of total RNA was used for each reaction of reverse transcription by using PrimeScript RT reagent Kit (Takara) and then subjected to real-time PCR using TB Green Premix Ex Taq II (Tli RNase H Plus) kits (Takara) on an Applied Biosystems 7500 Fast Real-Time PCR System (ABI). Each sample was performed in triplicate, and all results were normalized to the expression of being normalized to β-actin (ACTIN). Data was analyzed using the 2^–ΔΔCt^ method against β-actin. Results were expressed as fold changes compared to the control. The sequences of PCR primers were listed in Supplementary Table 1.

### Immunofluorescence Staining

Cells were fixed with 4% paraformaldehyde for 15 min at room temperature and permeabilized with 0.05% Triton X-100 for 10 min. Then, cells were stained with the following primary antibodies overnight at 4°C: α-SMA, Calponin, SM22α, CD31, VEGFR1, and VEGFR2. After washing with PBS for three times (5 min each), the cells were stained with secondary antibodies (Life Tech; Alexa Fluor 488) diluted 1:500 in PBS. Nuclei were stained with DAPI (Sigma). Images were acquired using a fluorescence confocal microscope (Leica).

### 5-Ethynyl-20-Deoxyuridine (EdU) Cell Proliferation Assay

Cells were seeded on a 24-well plate and cultured overnight. The 5-ethynyl-20-deoxyuridine agent (EdU, Beyotime, Shanghai, China) was added to each well and allowed 2 h for incorporation. The cells were fixed in 4% PFA for 20 min and washed three times with PBS containing 3% bovine serum albumin (BSA). The cells were incubated in PBS containing 0.3% Triton X-100 for 15 min, followed by BSA-containing PBS washes three times. The cells were stained with 5 μg/mL Hoechst 33342 at room temperature for 10 min and washed three times with PBS.

### CCK-8 Assay

Cells were counted 24 h after adhering to the 96-well plate. The original culture medium was replaced with 100 μL fresh culture medium containing 10 μL CCK-8 reagent (Beyotime, Shanghai, China) and continued to culture for 2 more hours. The optical density (OD) at 450 nm was measured using an automatic plate reader (Bio-Rad, United States), which was used to determine cell proliferation.

### Migration Assay

Migration of PVASCs was assessed by wound-healing scratch. Cells were seeded into six-well plates to confluence for 24 h. Then, an 800-μm tip was used to produce a uniform linear scratch in the center of the wells. The images of cell migration across the wound were captured at 0 h and 24 h using a microscope with a digital camera.

### Murine Femoral Artery Guidewire Injury Model and Cell Transplantation

The procedure used for this model is well-established based on our own previous work (Xie et al., 2017). Briefly, male mice were anesthetized with an intraperitoneal injection of pentobarbital sodium. An arteriotomy was performed in the epigastric branch of both sides of the femoral arteries. A 0.014’ guide wire (Hi-Torque, Cross IT 200 XT) was penetrated into the femoral artery and subsequently pulled back and forth three times, resulting in endothelial denudation. The femoral artery was ligated after the retreat of a guidewire. Sham injury without guidewire injury was regarded as negative control. All successful models were transplanted with PVASCs (sh-scramble, sh-TBX20, LV-NC, LV-TBX20). 1 × 10^6^ cells were suspended in 30 μL Matrigel, which was applied at the adventitial side of the injured femoral artery. Twenty-eight days after cell transplantation, the animals were sacrificed and histologically examined.

### Histology

The injured femoral arteries were carefully harvested 4 weeks post operation. Samples were subsequently embedded in paraffin and cut into 4-μm-thick sections. The sections were then stained with hematoxylin and eosin (H&E) to analyze the neointimal areas and intima/media (I/M) ratios.

### Single-Cell RNA-Sequencing Analysis

The single-cell RNA-seq dataset was obtained from Professor Pingjin Gao. Detailed information of this dataset can be found in the article named “Perivascular adipose tissue-derived stromal cells contribute to vascular remodeling during aging” (Pan et al., 2019). The Seurat V3 on RStudio was applied to perform single-cell analysis for cell clustering, differential gene expression analysis, and gene expression visualization. In terms of the analysis procedure, the data was filtered by standard Seurat functions of nFeature_RNA, nCount_RNA, and percent.mt (Supplementary Figures 2A–G). The data was then further refined by functions including NormalizeData(), FindVariableFeatures(), RunPCA(), RunTSNE(), and FindAllMarkers() in Seurat package. The data of single-cell analysis of the aorta was extracted from “Atheroprotective roles of smooth muscle cell phenotypic modulation and the TCF21 disease gene as revealed by single-cell analysis” from Nature Medicine 2 (GSE131778).

### RNA Sequence Analysis

Bulk mRNA sequencing was performed by Novogene company. Subcutaneous, abdominal, and perivascular adipose-derived stem cells at passages 3–6 were harvested for the following sequencing. Subsequent analysis was achieved by applying RStudio. Pearson analysis, box plot, PCA plot, and violin plot of FPKM were performed for quality control (Supplementary Figures 2H–K).

### Statistical Analysis

The number of replications applied in each respective experiment is displayed in each figure individually. All the results are presented as mean ± SEM. T-student test was used between two groups, and ANOVA was used among more than two experimental groups. Values of *P* < 0.05 were considered to be significant.

## Results

### TBX20 Expressed Highly in Perivascular Adipose-Derived Stem Cells

**FIGURE 1:**
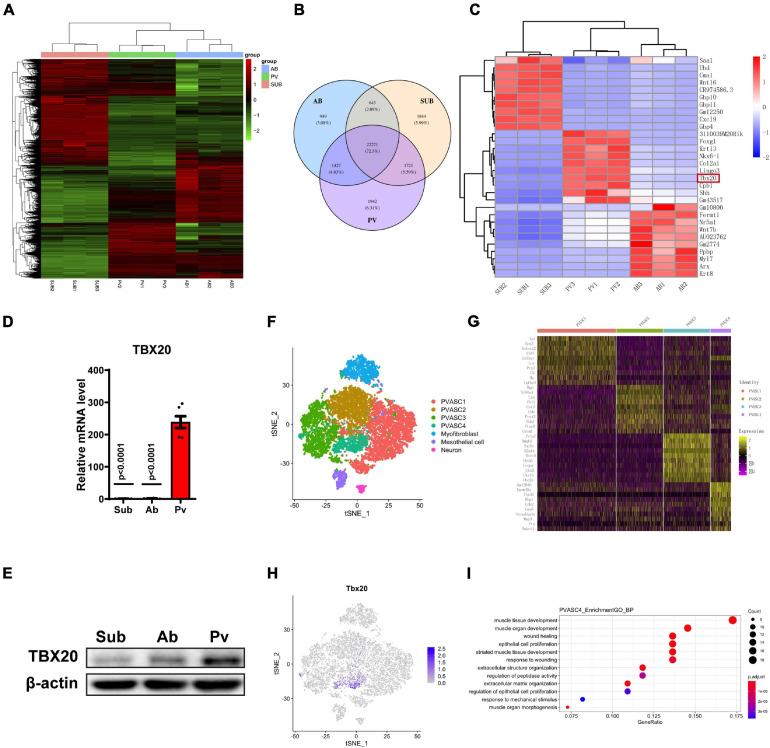
TBX20 expressed highly in perivascular adipose-derived stem cells. **(A,B)** Bulk mRNA sequencing of subcutaneous (Sub), abdominal (Ab), and perivascular (Pv) ASCs. **(C)** Top 10 differentially expressed genes among subcutaneous, abdominal, and perivascular ASCs. **(D)** QPCR of TBX20 among subcutaneous, abdominal, and perivascular ASCs. **(E)** Western Blot of TBX20 among subcutaneous, abdominal, and perivascular ASCs. **(F–I)** Single-cell RNA-seq predicts the effect of TBX20 on capacities of PVASCs. **(F)** T-SNE map showing four subpopulations of PVASCs. **(G)** Heatmap displayed the expression of top 10 differential genes for four clusters. **(H)** Individual t-SNE visualization of TBX20 displayed the respective TBX20^+^ subpopulation. **(I)** Geno ontology analysis represented the biological process for PVASC4.

Previous publications demonstrated the anatomical, physiological, cellular, clinical, and prognostic differences among adipose tissues present in subcutaneous areas, abdominal cavity, and outside the adventitial layer of artery (Rittig et al., 2012). Thus, we investigated the difference of ASCs from subcutaneous adipose tissue, abdominal adipose tissue, and perivascular adipose tissue at transcriptional expression. Here, we performed bulk RNA-Seq assay for the above three kinds of ASCs. A heatmap of differentially expressed genes (DEGs) indicated significant variances among PVASCs, Ab-ASCs, and Sub-ASCs (Figure 1A). A total of 1,942 genes were expressed particularly in PVASCs, 949 genes in AB-ASCs, and 1,844 genes in Sub-ASCs (Figure 1B). The top 10 most distinct genes were plotted in the heatmap (Figure 1C). Among all DEGs, TBX20 raised our interest, which was highly expressed in PVASCs and previously reported to be involved with endothelial development and angiogenesis (Meng et al., 2018). Quantitative real-time polymerase chain reaction (Figure 1D) and Western Blot (Figure 1E) validated such unique higher expression of TBX20 in PVASCs.

### TBX20 May Be Involved in the Differentiation of PVASCs to SMCs and ECs

To further explore the characteristics of PVASCs, we analyzed single-cell RNA-seq data of PVASCs from a previous publication (Pan et al., 2019). The T-SNE map showed four subpopulations of PVASCs (Figure 1F and Supplementary Figure 3A). The heatmap of the top 10 DEG further displayed PVASCs’ heterogeneity, among which TBX20 was uniquely expressed in PVASCs’ subpopulation 4 (Figures 1G,H). GO analysis further inferred that PVASC4 was the only subpopulation involved in both muscle development and epithelial cell proliferation, all of which contributed to vascular remodeling (Figure 1I and Supplementary Figures 3B–D). The Panther pathway confirmed that TGF-beta, FGF, Wnt, and integrin signaling pathways were among the top five correlated pathways (Supplementary Figure 3E), all of which were closely related to vascular remodeling.

**FIGURE 2:**
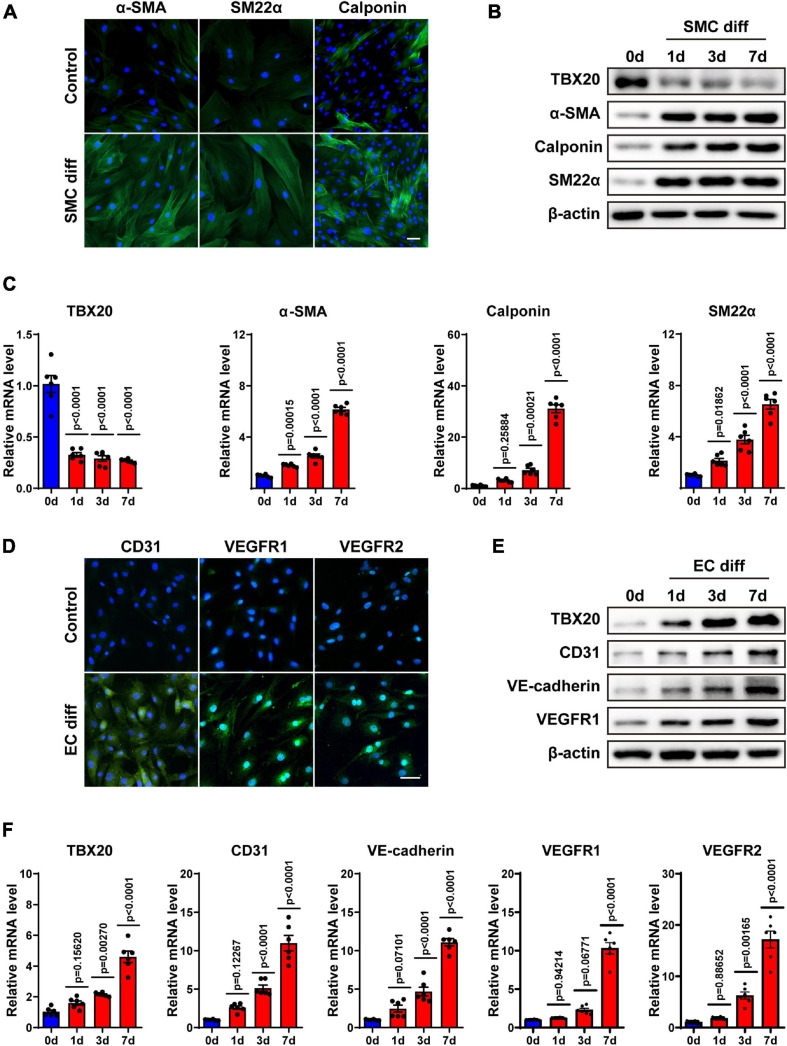
TBX20 may play a role in the differentiation of PVASCs to SMCs and ECs. **(A)** PVASCs were treated with or without TGF-β1 (5 ng/mL) for 7 days. Immunofluorescent staining showed the upregulation of SMC markers. **(B,C)** PVASCs were cultured in SMC differentiation medium for 0, 1, 3, and 7 days. Western Blot analysis and QPCR analysis of TBX20, α-SMA, SM22α, and calponin. **(D)** PVASCs were cultured in the control medium or EGM-2 medium for 7 days. Immunofluorescent staining of EC markers showed upregulation in differentiated PVASCs toward endothelial cells. **(E,F)** PVASCs were cultured in the EGM-2 medium for 0, 1, 3, and 7 days. Western Blot analysis and QPCR analysis of TBX20, CD31, VE-cadherin, VEGFR1, and VEGFR2. Scale bar = 100 μm. Cell nuclei were stained with DAPI.

Taking the analysis results and previous publications together, we hence doubted whether TBX20 regulated EC or SMC differentiation of PVASCs. To confirm this hypothesis, PVASCs were treated with TGF-β1 (5 ng/mL) for 1, 3, and 7 days to induce SMC differentiation as previously described (Gu et al., 2019). SMC differentiation was confirmed by immunofluorescence staining (Figure 2A). As shown in Figures 2B,C, both the protein and mRNA levels of SMC differentiation-specific markers, α-SMA, SM22, and Calponin, were significantly increased in response to TGF-β1 with the decreased TBX20 expression in a time-dependent manner.

For EC differentiation, PVASCs were induced in the EGM-2 medium for 1, 3, and 7 days. To confirm the endothelial phenotype, the induced cells were harvested and analyzed for EC-specific markers (CD31, VEGFR1, VEGFR2) by immunofluorescence staining (Figure 2D). Real-time PCR and Western Blot showed that levels of EC markers such as CD31, VE-cadherin, VEGFR1, and VEGFR-2 were significantly increased in the differentiated cells (Figures 2E,F). Along with EC markers, in contrast to SMC differentiation, expression of TBX20 was upregulated during EC differentiation.

The above data proved that PVASCs could give rise to vascular lineages *in vitro*, establishing a theoretical foundation for its participation in vascular remodeling *in vivo*. Interestingly, the expression of TBX20 was opposite in SMC and EC differentiation from PVASCs, which drove us to the following experiments.

### Knockdown of TBX20 Improved SMC Differentiation and Inhibited EC Differentiation

**FIGURE 3:**
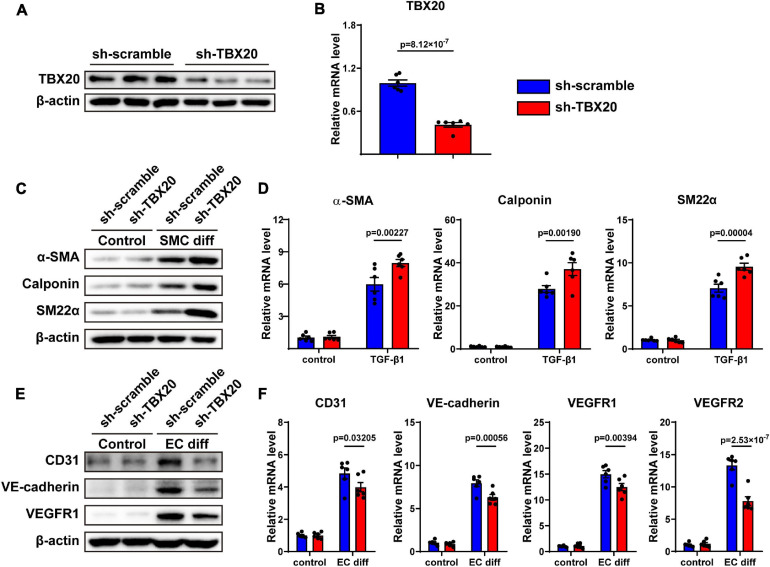
Knockdown of TBX20 improved SMC differentiation and inhibited EC differentiation. **(A,B)** TBX20 level was significantly downregulated after shRNA TBX20 transfection, determined with Western Blot **(A)** and QPCR **(B)**. **(C,D)** Transfection of PVASCs with sh-TBX20 lentivirus improved the level of SMC markers as shown by Western Blot **(C)** and QPCR **(D)**. **(E,F)** Transfection of PVASCs with sh-TBX20 lentivirus inhibited the level of EC markers as shown by Western Blot **(E)** and QPCR **(F)**.

To determine whether TBX20 regulated cell differentiation, PVASCs were infected with lentivirus-expressing shRNA sequences of TBX20 or sh-scramble (control) sequence. The efficiency of knockdown for TBX20 was confirmed by Western Blot and QPCR analysis (Figures 3A,B). We then treated control (sh-scramble) and TBX20 knockdown (sh-TBX20) PVASCs with TGF-β1 to induce SMC differentiation. Both mRNA and protein levels of α-SMA, SM22α, and Calponin were markedly increased in sh-TBX20 PVASCs (Figures 3C,D), indicating that TBX20 knockdown promoted SMC differentiation. On the contrary, after EC differentiation for 7 days, the mRNA levels and protein expressions of EC markers (CD31, VE-cadherin, VEGFR1 and VEGFR2) significantly declined upon the inhibition of TBX20 (Figures 3E,F).

To further investigate whether TBX20 was involved in other cell biological processes, we also performed assays for cell migration and proliferation, both of which played crucial roles in vascular remodeling. However, we found that knockdown of TBX20 did not affect migration and proliferation of PVASCs (Supplementary Figures 4A–E). In summary, TBX20 acted oppositely and regulated the cell differentiation of both SMC and EC from PVASCs.

### Overexpression of TBX20 Suppressed SMC Differentiation and Promoted EC Differentiation

**FIGURE 4:**
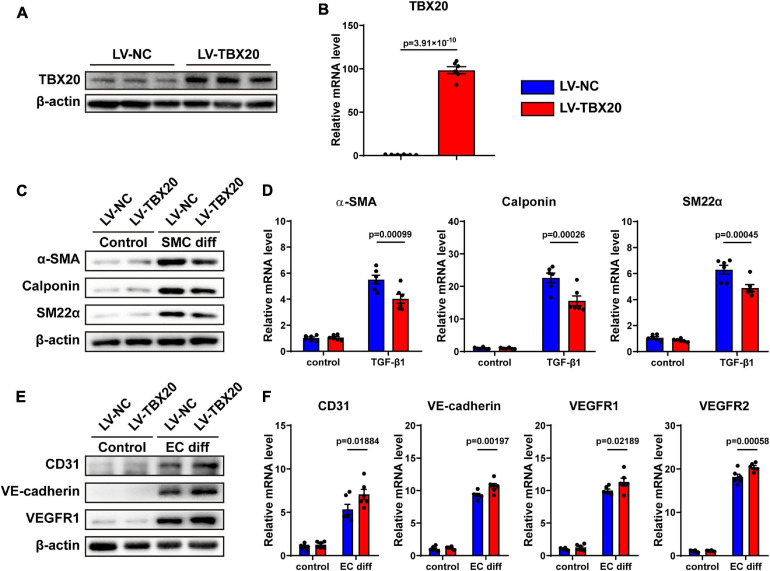
Overexpression of TBX20 suppressed SMC differentiation and promoted EC differentiation. **(A,B)** TBX20 level was significantly upregulated after LV-TBX20 lentivirus transfection, determined with Western Blot **(A)** and QPCR **(B**). **(C,D)** Transfection of PVASCs with LV-TBX20 lentivirus suppressed the level of SMC markers as shown by Western Blot **(C)** and QPCR **(D)**. **(E,F)** Transfection of PVASCs with the LV-TBX20 lentivirus promoted the level of EC markers as shown by Western Blot **(E)** and QPCR **(F)**.

To further investigate whether TBX20 was involved in SMC and EC differentiation, PVASCs were transfected by the TBX20-overexpressing lentivirus vector and control vector. Compared with the LV-NC group, both mRNA and protein levels of TBX20 were significantly increased in the LV-TBX20 group (Figures 4A,B). After TGF-β1 stimulation, QPCR and Western Blot analyses revealed that overexpressing TBX20 significantly suppressed SMC gene expressions (Figures 4C,D). After PVASCs were induced with the EGM-2 medium for endothelial differentiation for 7 days, a higher expression of EC markers was detected in the LV-TBX20 PVASC group compared with the LV-NC group (Figures 4E,F).

Taken together, these findings suggested that TBX20 suppressed SMC differentiation and promoted EC differentiation. In addition, similar to previous results, overexpression of TBX20 did not affect the migration and proliferation of PVASCs (Supplementary Figures 5A–E). It is interesting that TBX20 could regulate both EC and SMC differentiation where the trends of TBX20 expression are totally inverse during cell differentiation, respectively.

### TBX20 Was Important for the Activation of Smad2/3 Signaling During SMC Differentiation

**FIGURE 5:**
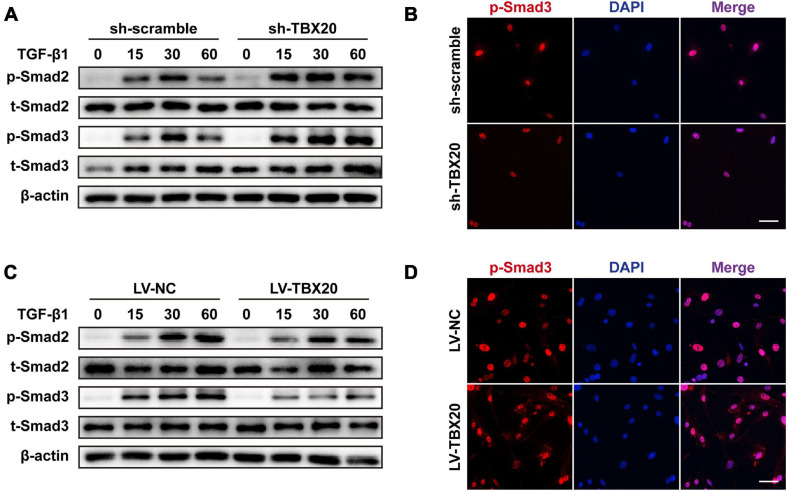
TBX20 was important to the activation of Smad2/3 signaling during SMC differentiation. **(A,C)** PVASCs were incubated with 5 ng/mL TGF-β1 after starving with 2% FBS medium for 24 h, and the samples were harvested at 15, 30, and 60 min. Western Blot analyses detected the phosphorylation of Smad2/3. **(B,D)** Immunofluorescence staining revealed that more Smad3 was translocated into the nuclei in the sh-TBX20 cells and less Smad3 was translocated into the nuclei in the LV-TBX20 cells. Scale bar = 50 μm. Cell nuclei were stained with DAPI.

Hinted by bioinformatic analysis, TBX20^+^ PVASC4 was the only subpopulation responsible for muscle development. We hence explored the underlying mechanism of TBX20 in PVASC differentiation toward SMCs. Control (sh-scramble) and TBX20-knockdown (sh-TBX20) PVASCs were starved for 24 h in the serum-free medium and subsequently treated with TGF-β1 for 15, 30, and 60 min prior to collection for Western Blot of Smad phosphorylation. TBX20 knockdown significantly increased the phosphorylation of Smad2/3 in response to TGF-β1 (Figure 5A). To further determine the role of TBX20 in Smad activation, we examined Smad2/3 nucleus translocation responses to TGF-β1 by immunofluorescence staining in control or TBX20-knockdown cells. We confirmed that Smad2/3 could translocate into the nuclei after TGF-β1 treatment in control cells, and Smad2/3 nuclear translocation was significantly enhanced in TBX20-knockdown cells (Figure 5B). On the other hand, after TBX20 overexpression, phosphorylation and nuclear translocation of Smad2/3 were reduced, compared with the LV-NC group (Figures 5C,D). These suggested a potential mechanism for TBX20 as an effect mediator in regulating Smad2/3 signaling.

### Role of TBX20 in Neointimal Hyperplasia After Vascular Injury

**FIGURE 6:**
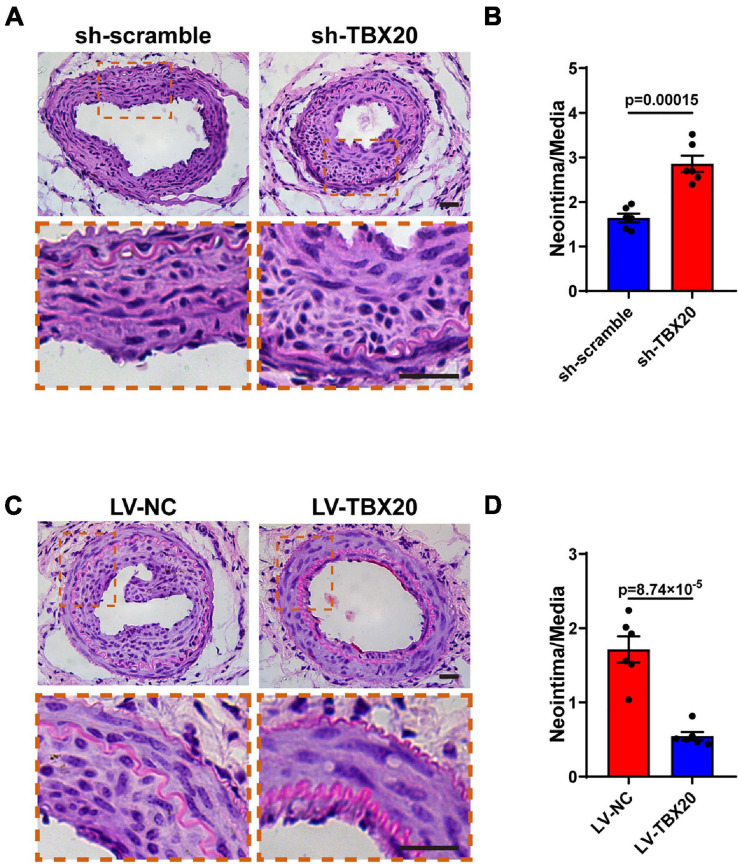
Role of TBX20 in neointimal hyperplasia after vascular injury. **(A,C)** H&E staining of injured femoral arteries at 28 days post PVASCs transplantation. **(B,D)** Quantitative analysis of the area ratio of neointima to media. Scale bar = 50 μm.

Firstly, to exclude the role of TBX20 in other vascular lineage cells, we analyzed single-cell sequencing data of the aorta. Our analysis revealed that all cell types within the aorta hardly expressed TBX20 (Supplementary Figures 6B–E). We also performed QPCR for PVASCs, vascular SMCs, and vascular tunica media, thus excluding the role of TBX20 in resident SMCs (Supplementary Figure 6A). To investigate the role of TBX20 in PVASCs in neointimal hyperplasia, we performed guidewire injury of the femoral artery in mice. Male mice underwent guidewire injury of the femoral artery, followed by transplantation of PVASCs transfected with sh-TBX20 lentivirus or sh-scramble lentivirus, LV-TBX20 lentivirus, or LV-NC lentivirus in Matrigel at the adventitial side of the injured vessel. Four weeks postsurgery, vascular histological staining indicated that TBX20 knockdown promoted intimal hyperplasia. Compared with the sh-scramble group, the intima/media ratio in the sh-TBX20 group was increased (Figures 6A,B). In the transplantation of LV-TBX20 PVASCs after vascular injury, neointima formation was significantly inhibited. Compared with the LV-NC group, the intima/media ratio in the LV-TBX20 group was greatly reduced (Figures 6C,D). Altogether, these findings indicated that TBX20 played a crucial role in neointima hyperplasia in response to endovascular injury.

## Discussion

Adipose tissues are present at multiple locations. Adipose tissues at different locations are distinct in morphology and composition (Wu et al., 2012). Perivascular adipose tissue (PVAT) draws a lot of attention because of its anatomic location and environmental/metabolic context (Fitzgibbons et al., 2011; Chang et al., 2012, 2020; Padilla et al., 2013). PVAT harbors mesenchymal stem cells, which can be a promising source for stem cell therapies. In the present study, we performed RNA-Seq assay to measure the gene expression of ASCs from subcutaneous, abdominal, and perivascular adipose tissue. Among the top 10 genes specifically expressed in PVASCs, transcriptional factor TBX20 is the only one which had been proved closely related to vascular angiogenesis (Shen et al., 2013; Meng et al., 2018). With the permission of Professor Pingjin Gao, we reanalyzed her single-cell data of PVASCs and identified a TBX20^+^ subpopulation of PVASCs. Such finding and subsequent GO analysis further consolidated our confidence of TBX20’s role in cell differentiation and vascular remodeling.

Many studies showed the roles of TBX20 regulating cardiac development (Meins et al., 2000; Stennard et al., 2005; Kirk et al., 2007). Few evidences addressing the vascular system indicated that TBX20 mediated angiogenic processes and suppressed endothelial cell injury (Shen et al., 2013; Meng et al., 2018). Moreover, TBX20 overexpression enhanced cardiomyogenic differentiation and inhibited osteogenic differentiation in human adipose-derived MSCs (Neshati et al., 2018; Mollazadeh et al., 2019). In the present study, we demonstrate for the first time that TBX20 simultaneously mediates both SMC and EC differentiation of PVASCs *in vitro* and modulates the neointimal hyperplasia after vascular injury in mice. Our study revealed that TGF-β1, which is of great potency for SMC differentiation (Chen and Lechleider, 2004), significantly downregulated TBX20 expression during PVASC differentiation into SMCs. Phosphorylation of Smad2/3 is one of the canonical pathways in TGF-β1-induced SMC differentiation (Derynck and Zhang, 2003). In our study, we found that TBX20 inhibited the phosphorylation and nucleus translocation of Smad2/3 in response to TGF-β1, suppressing SMC-specific gene expression in PVASCs. Moreover, TBX20 displayed opposite effects on differentiation of PVASCs to ECs and SMCs. TBX20 significantly increased during the differentiation of PVASCs into endothelial cells and TBX20 promoted EC differentiation. Such data suggested that TBX20 might act as a buffer and balance cell fates. Maintaining an appropriate level of TBX20 expression could be meaningful for maintaining homeostasis in vascular remodeling.

PVASCs can differentiate to SMCs, and cell transplantation assays confirmed their involvement in neointimal hyperplasia through migration from the adventitia side to the intima and SMC differentiation (Gu et al., 2019). In the present study, we tested the effects of TBX20 on intimal hyperplasia by means of cell transplantation in the murine femoral artery guidewire injury model. Our data showed that TBX20 overexpression in PVASCs through genetic modification significantly inhibited neointimal proliferation. Moreover, transplantation of TBX20-knockdown PVASCs increased the neointimal hyperplasia. There is still a debate on how mesenchymal stem cells participate in various pathological processes. Cell differentiation, cytokine secretion, and immunomodulation are three mainstream hypotheses. Underlying mechanism of PVASCs participation in neointiaml formation requires additional work. Many strategies were implemented for the prevention of vascular stenosis. For instance, sirolimus-eluting stents have been widely used in coronary artery diseases which can prevent occlusion by inhibiting SMC proliferation (Suzuki et al., 2001). However, in-stent restenosis is still of common occurrence, implying that SMCs should not be the only one to blame. Here we provide a potential therapeutic target for vascular stenosis diseases independent of SMCs, vascular adventitial stem cells, and macrophages.

In conclusion, our study demonstrated that TBX20 could inhibit SMC differentiation and promote EC differentiation *in vitro*. *In vivo*, TBX20 could effectively inhibit neointima formation after vascular injury, which provided a new intervention target for vascular diseases like restenosis.

## Data Availability Statement

The data presented in the study are deposited in the (GEO) repository, accession number (GSE171946).

## Ethics Statement

The animal study was reviewed and approved by Animal Ethics Committee of Zhejiang University.

## Author Contributions

YJ, YM, and JS performed the research and drafted the manuscript. HN and YL carried out the cell culture. YuZ and HM participated in the statistical analysis. CL, YiZ, and SD participated in the histological analysis. MX and YX conceived the study and participated in the study design. All authors read and approved the final manuscript.

## Conflict of Interest

The authors declare that the research was conducted in the absence of any commercial or financial relationships that could be construed as a potential conflict of interest.

## Abbreviations

PVASCs: perivascular adipose-derived stem cells
SMCs: smooth muscle cells
ECs: endothelial cells
H&E: hematoxylin–eosin
PVAT: perivascular adipose tissue
α-SMA: smooth muscle α-actin
SM22α: smooth muscle 22 alpha
TGF-β1: transforming growth factor-β1
VEGFR1: vascular endothelial growth factor receptor 1
VEGFR2: vascular endothelial growth factor receptor 2
VEGF: vascular endothelial growth factor
FGF: fibroblast growth factor
EGF: epidermal growth factor
IGF-1: insulin-like growth factor
BSA: bovine serum albumin
CCK8: Cell counting kit-8
PVDF: polyvinylidene difluoride.
