# Supplementary material for: TBX20 Contributes to Balancing the Differentiation of Perivascular Adipose-Derived Stem Cells to Vascular Lineages and Neointimal Hyperplasia
Source: Front Cell Dev Biol. 2021 Jun 2;9:662704. doi: 10.3389/fcell.2021.662704 (PMC8206642; doi:10.3389/fcell.2021.662704)
Supplement: Supplementary file 1 [file Image_1.PDF]

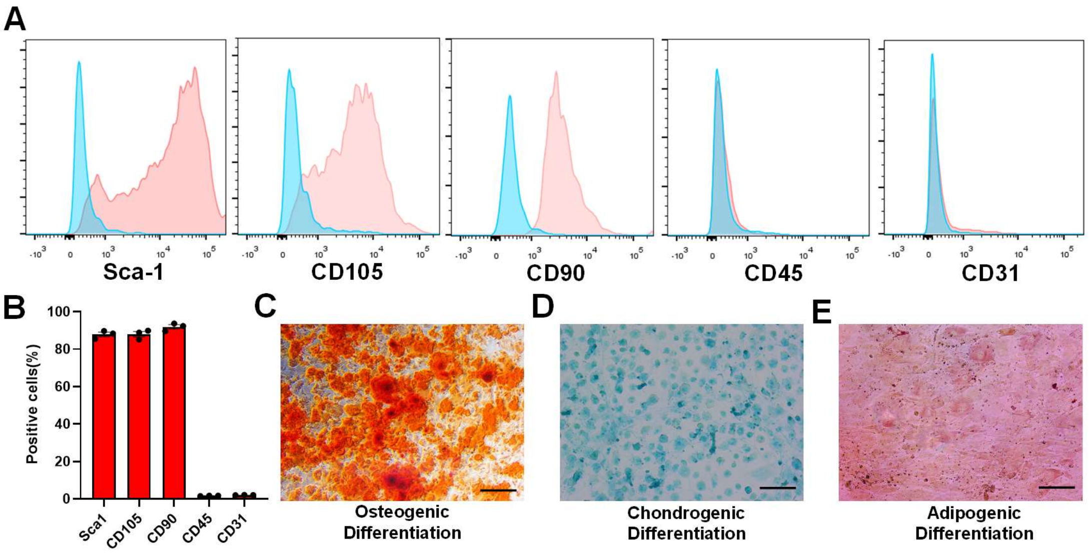

Supplementary Figure.1

A, Immunophenotype characterization of PVASCs by flow cytometry. **B,** Quantitative analysis of flow cytometry data. **C-E,** PVASCs showed differentiation capacities osteoblasts (**C**), chondroblasts(**D**) and adipocytes (**E**) by specific staining. Scale bar = 200 μm

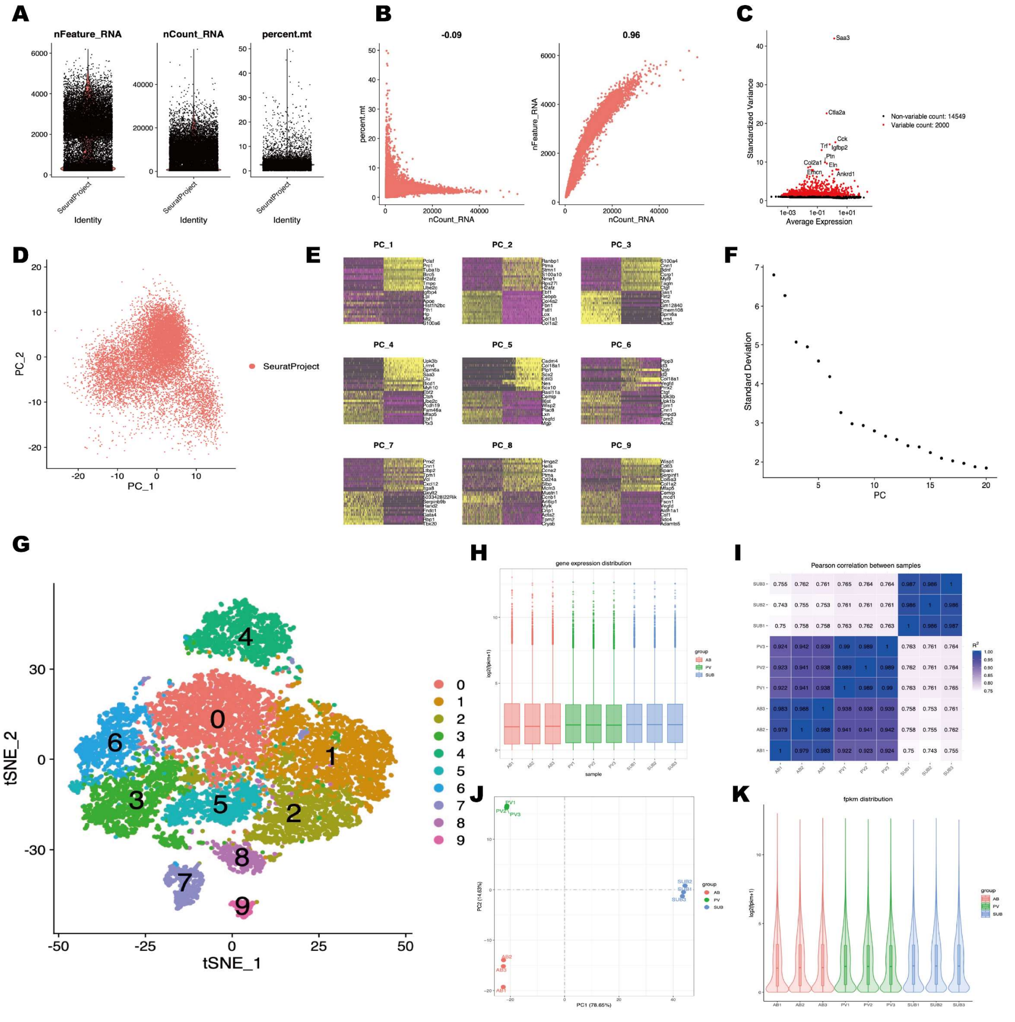

Supplementary Figure.2

A-B, Violin (**A**) and Scatter (**B**) visualization shows QC metrics and filtering basis. **C,** Variable features plot with labels of 10 most highly variable genes. Red dots correspond to 2,000 highly variable features used in downstream analysis. **D-E,** Dim plot (**D**) and heat map (**E**) Visualization shows principal component analysis (PCA) definition of both cells and features. **F,** Elbow plot shows a ranking of principle components (PCs) and determines the dimensionality of the dataset. **G,** T-SNE representation demonstrates 10 individual clusters. **H-K,** Box plot (**H**), Pearson correlation map (**I**), PCA plot (**J**) and violin plot (**K**) shows FPKM results of gene expression quantitative analysis. **FPKM**, expected number of Fragments Per Kilobase of transcript sequence per Millions base pairs sequenced. **t-SNE**, T-Distributed Stochastic Neighbor

Embedding.

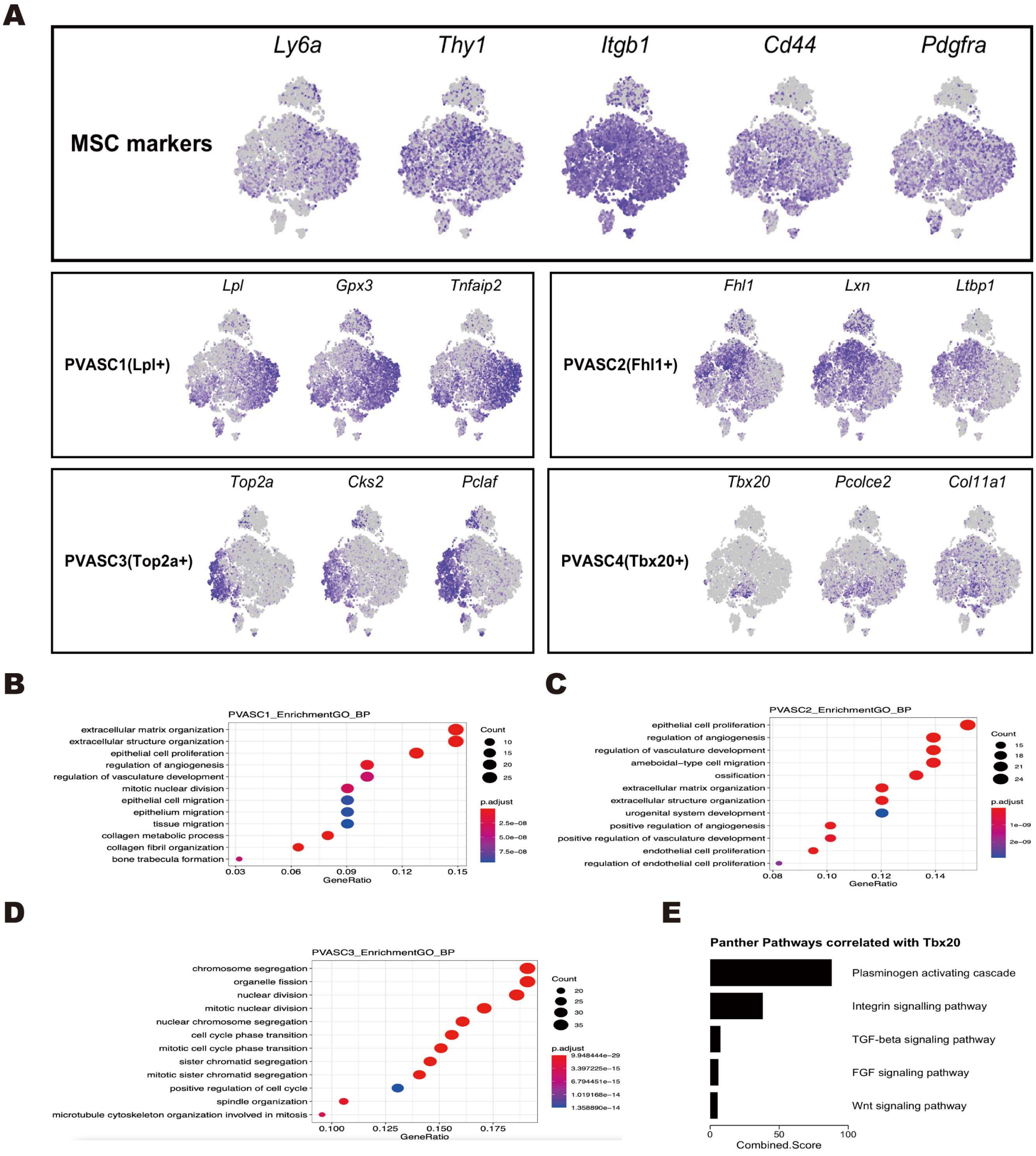

Supplementary Figure.3

A, Feature plot of MSC markers and top3 marker genes for PVASCs subpopulations. **B to D,** Gene ontology analysis represented the biological process for PVASC1 (**B**), PVASC2 (**C**), PVASC3 (**D**). **E,** Panther pathway of TBX20 correlated genes.

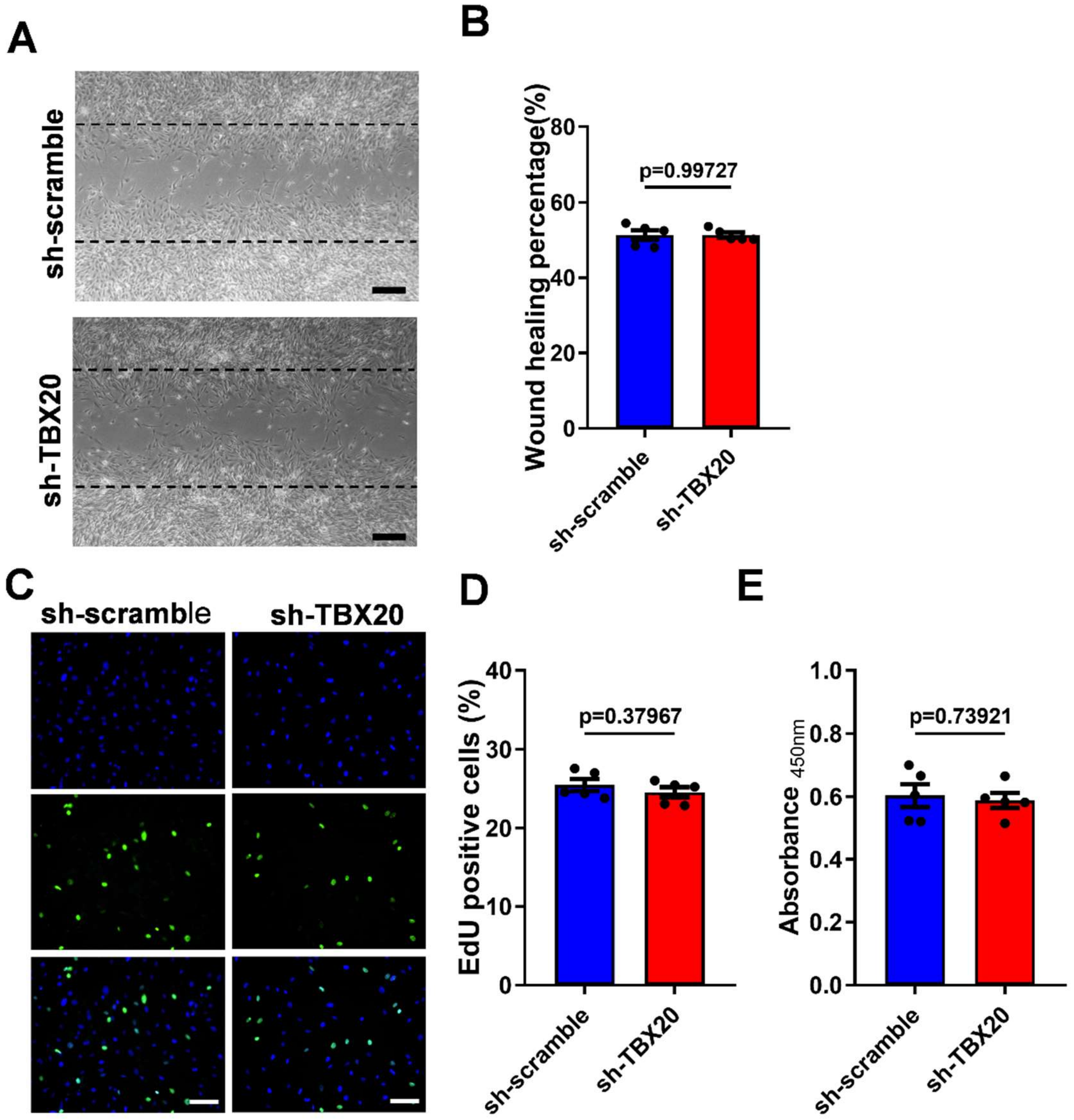

Supplementary Figure.4

A, Representative images showing the migration of sh-scramble and sh-TBX20 PVASCs was measured by wound-healing migration assay. Scale bar = 200 μm **B,** Quantitative analysis of migration percentage of sh-scramble and sh-TBX20 PVASCs 24 h after scraping. **C,** The proliferation of PVASCs was measured by EdU staining. Green indicated positive staining for EdU. Cell nuclei were stained with DAPI. Scale bar = 100 μm. **D,** The proliferation rate was quantified by the percentage of cells positive for EdU. **E,** In CCK8 assay, the proliferation rate of PVASCs was measured by Optical density (OD) at 450 nm.

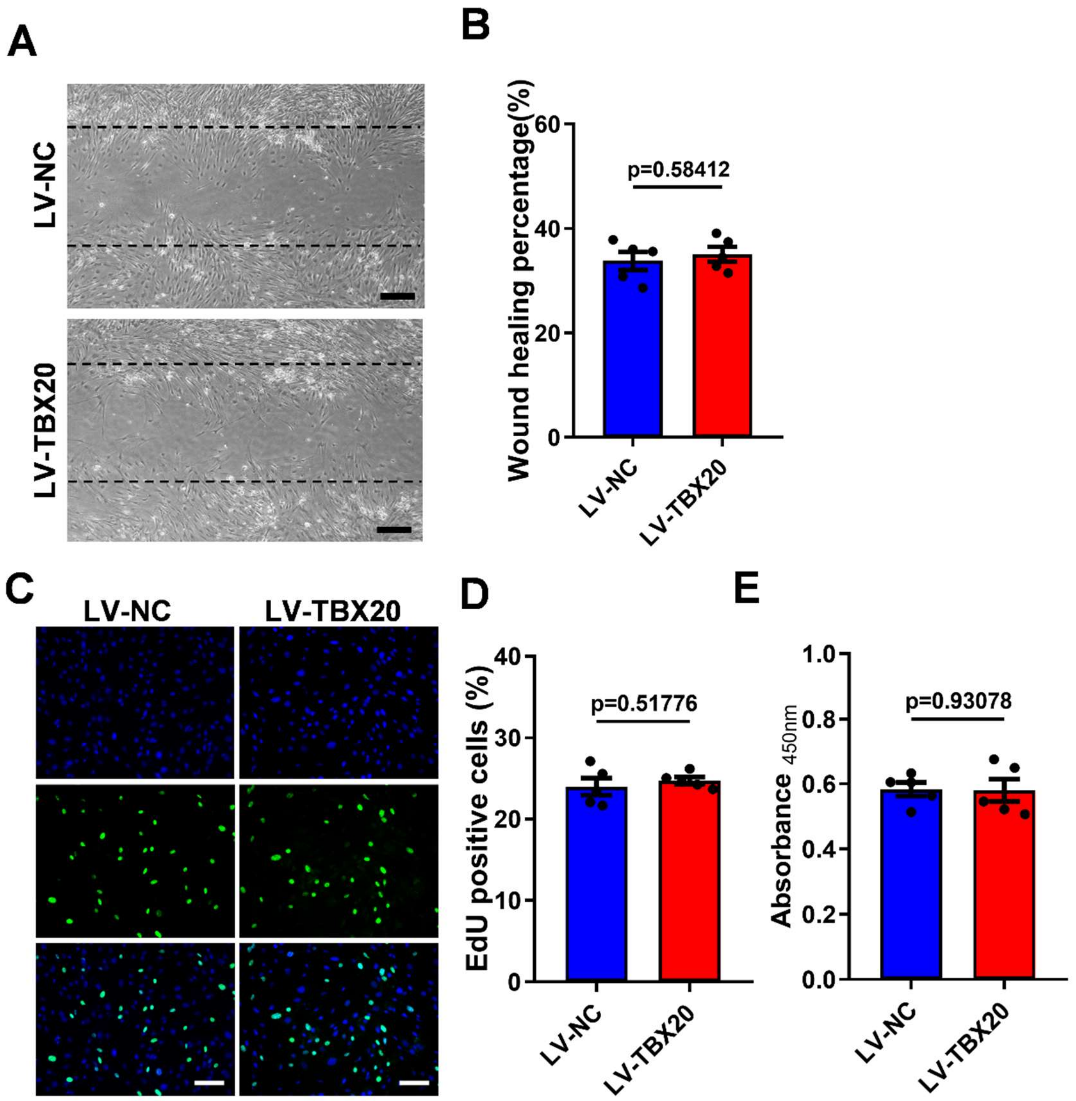

Supplementary Figure.5

A, Representative images showing the migration of LV-NC and LV-TBX20 PVASCs was measured by wound-healing migration assay. Scale bar = 200 μm. **B,** Quantitative analysis of migration percentage of LV-NC and LV-TBX20 PVASCs 24 h after scraping. **C,** The proliferation of PVASCs was measured by EdU staining. Green indicated positive staining for EdU. Cell nuclei were stained with DAPI. Scale bar = 100 μm. **D,** The proliferation rate was quantified by the percentage of cells positive for EdU. **E,** In CCK8 assay, the proliferation rate of PVASCs was measured by Optical density (OD) at 450 nm.

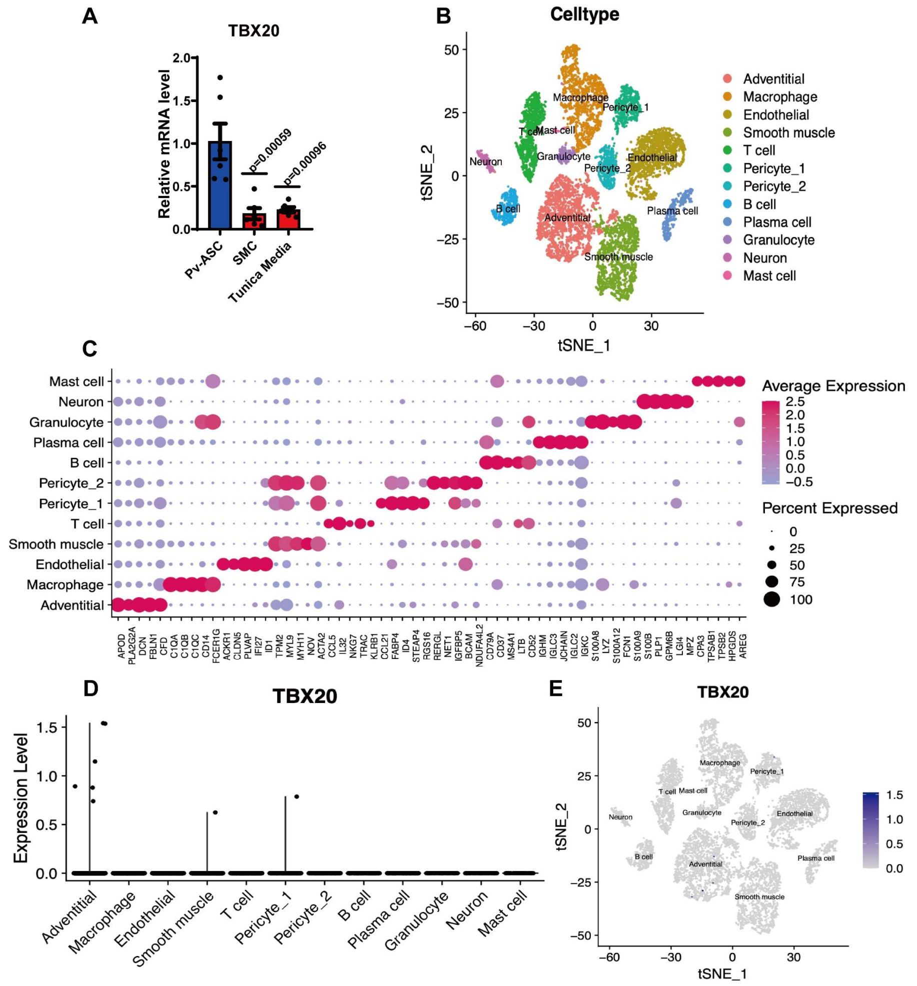

Supplementary Figure.6

A, QPCR of TBX20 in PVASCs, vascular SMC and vascular tunica media. **B,** T-SNE visualization of single-cell RNA sequencing data for the 12 identified aortic cell types. **C,** Dot plot of top markers in each aortic cell type. **D,** Violin plot of normalized TBX20 transcript expression values in each cell type. **E,** Feature plot of TBX20 expression.

Supplementary Table1 Primer list

Gene	Forward primer (5′-3′)	Reverse primer (5′-3′)
TBX20	AAACCCCTGGAACAATTTGTGG	CATCTCTTCGCTGGGGATGAT
α-SMA	TCCTGACGCTGAAGTATCCGAT	GGCCACACGAAGCTCGTTATAG
SM22α	GATATGGCAGCAGTGCAGAG	AGTTGGCTGTCTGTGAAGTC
Calponin	GGTCCTGCCTACGGCTTGTC	TCGCAAAGAATGATCCCGTC
CD31	GGACTCACGCTGGTGCTCTA	AGGAATGACGTAGCTCTCGG
VE-cadherin	GTGAACCGCCAGAATGCTAA	CTCTGGCACAGATGCGTTGA
VEGFR1	CTGCGACCCTCTTTTGGCTC	CAGTCTCTCCCGTGCAAACT
VEGFR2	CTCTGTGGTTCTGCGTGGAG	CGCTGTCCCCTGCAAGTAAT
